# Supplementary material for: Exploring the Potential of Sulfonamide-Dihydropyridine Hybrids as Multitargeted Ligands for Alzheimer’s Disease Treatment
Source: Int J Mol Sci. 2023 Jun 4;24(11):9742. doi: 10.3390/ijms24119742 (PMC10253445; doi:10.3390/ijms24119742)
Supplement: Supplementary file 1 [file ijms-24-09742-s001.zip › ijms-2353690-supplementary.pdf]

SUPPORTING INFORMATION

Exploring the Potential of Sulfonamide-Dihydropyridine Hybrids as Multitargeted Ligands for Alzheimer's Disease Treatment

Imen Dakhlaoui [1,2], Paul J. Bernard [1], Diana Pietrzak [3], Alexey Simakov [4], Maciej Maj [3], Bernard Refouvelet [1], Arnaud Béduneau [4], Raphaël Cornu [4], Krzysztof Jozwiak [3], Fakher Chabchoub [2], Isabel Iriepa [5], Helene Martin [4], José Marco-Contelles [6,*] and Lhassane Ismaili [1,*]

NMR Spectra for compounds 2a-c, 3a-c and 4a-i……………………………………….2-15

Experimental procedure of Inhibition of EeAChE and eqBuChE……………………16

Chromatograms of compound 4a and 4f…………………………………………………….17

^{1}H NMR spectrum of compound **2a**

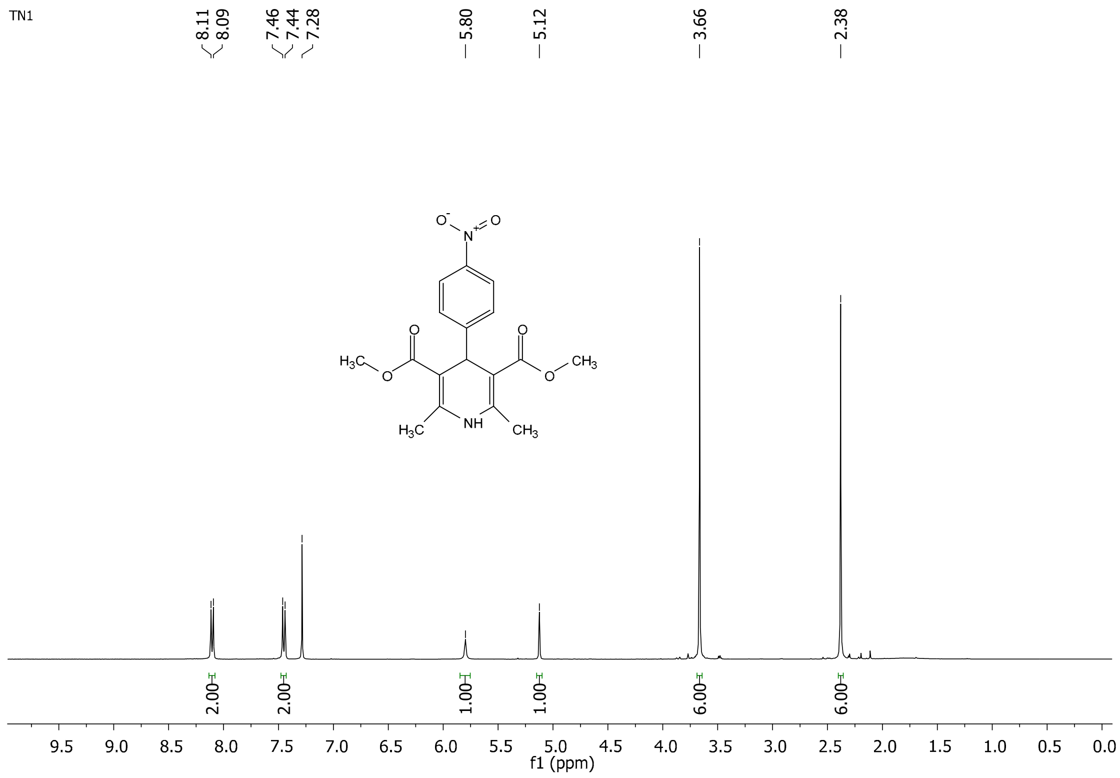

^{1}H NMR spectrum of compound **2b**

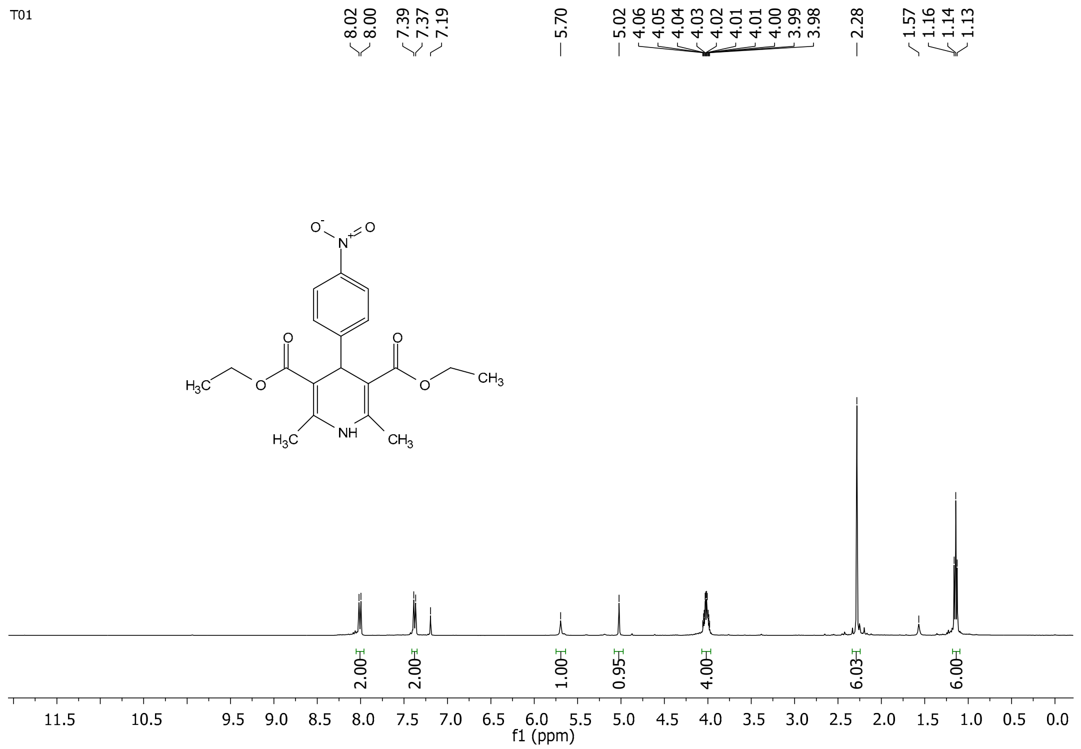

^{1}H NMR spectrum of compound **2c**

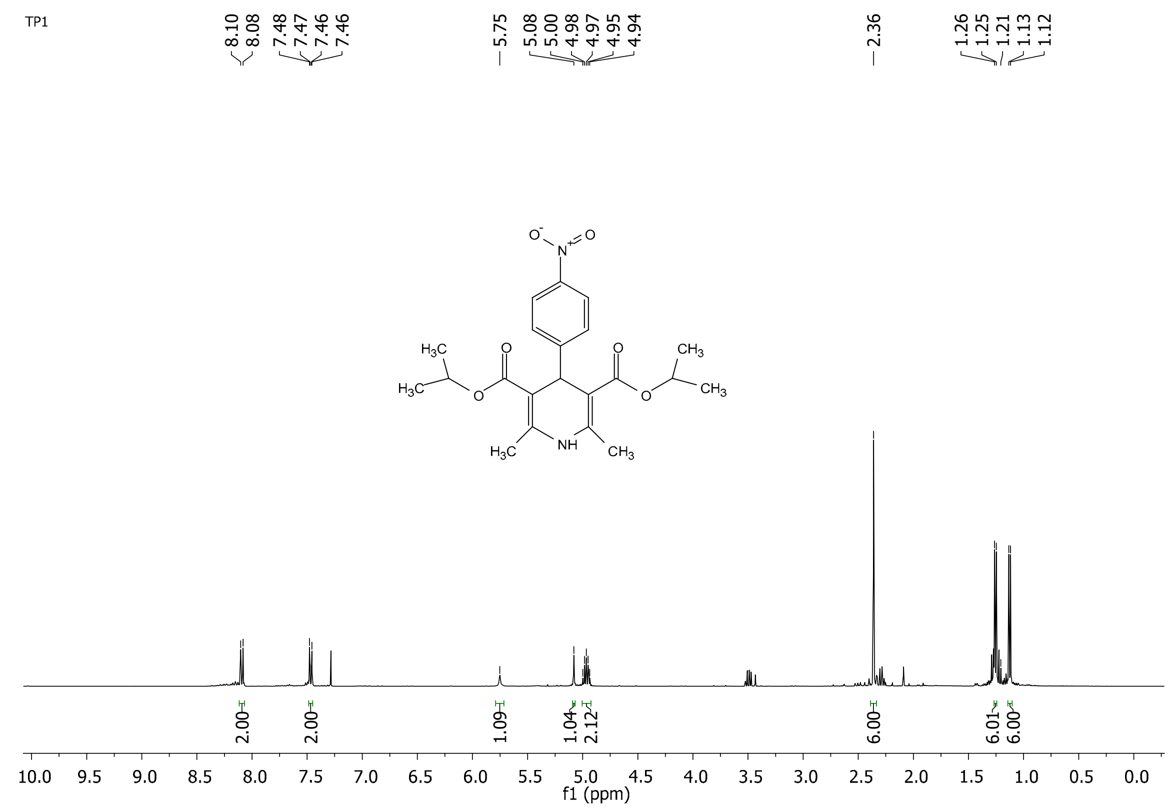

^{13}C NMR spectrum of compound **2c**

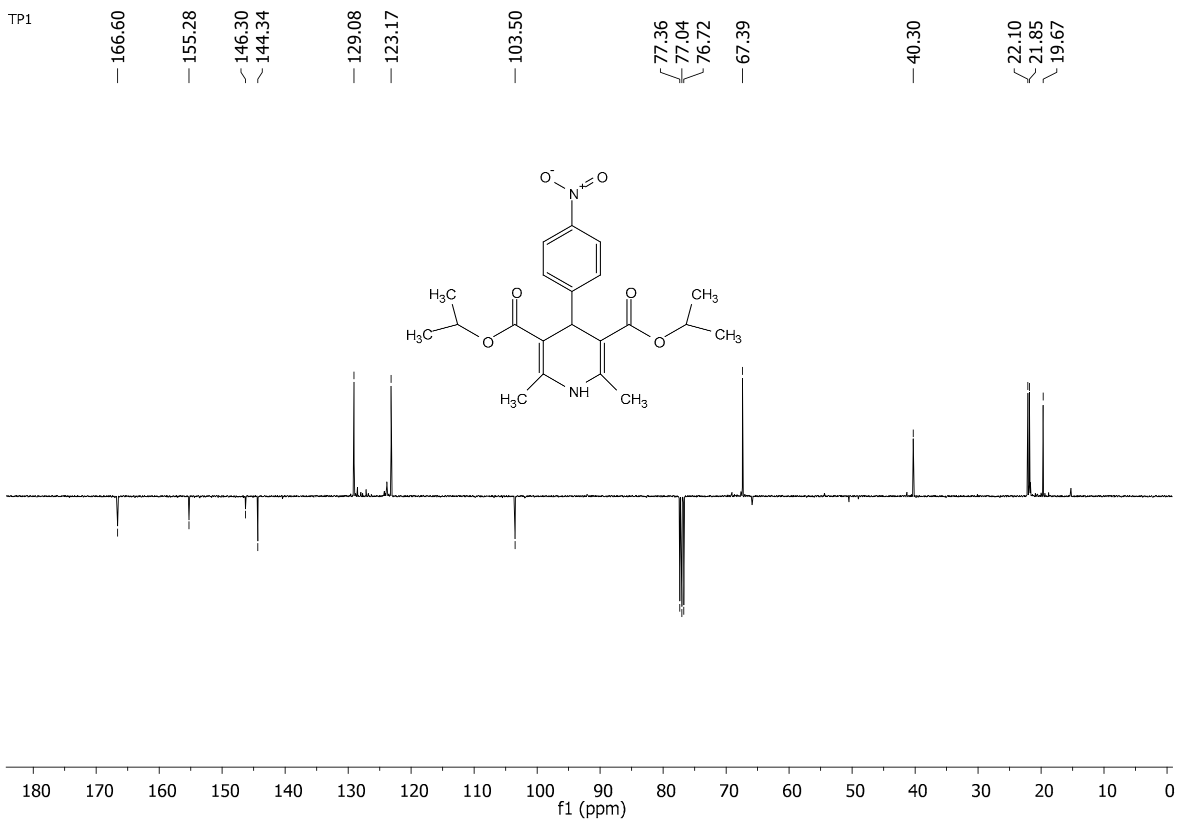

^{1}H NMR spectrum of compound **3a**

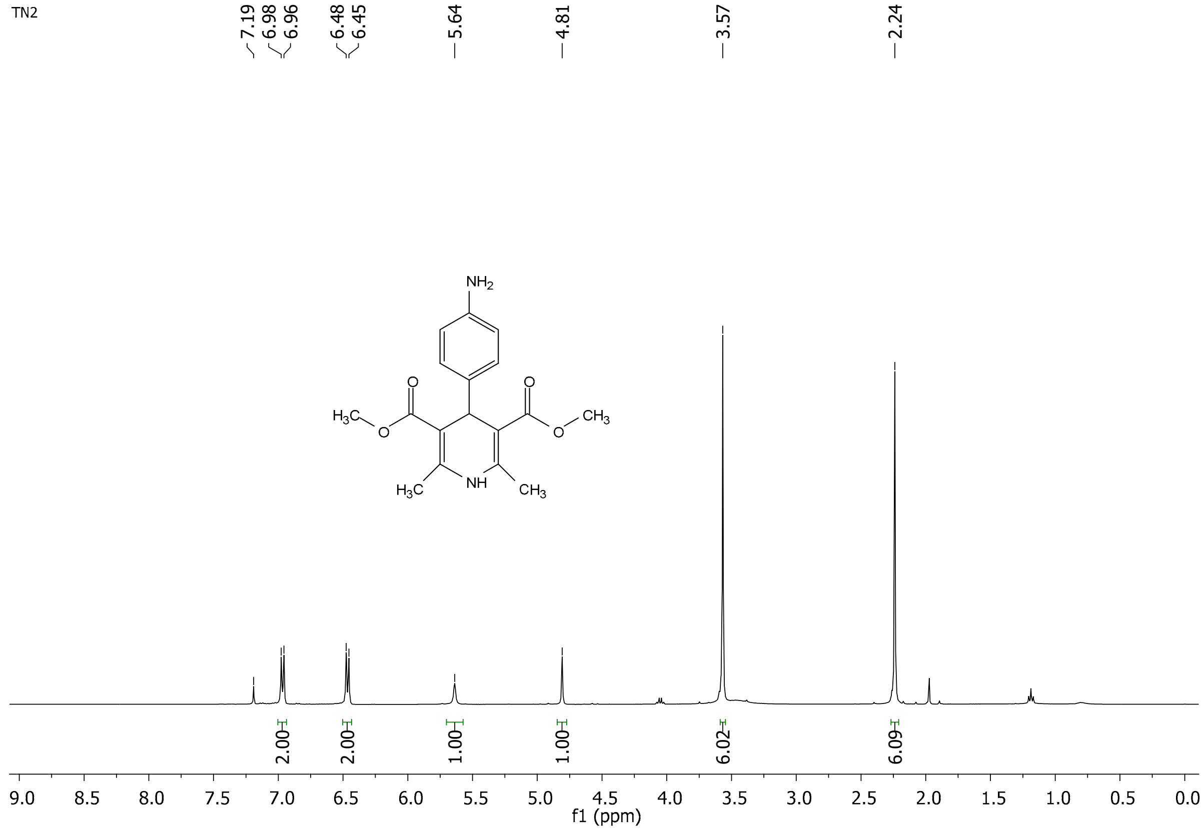

^{13}C NMR spectrum of compound **3a**

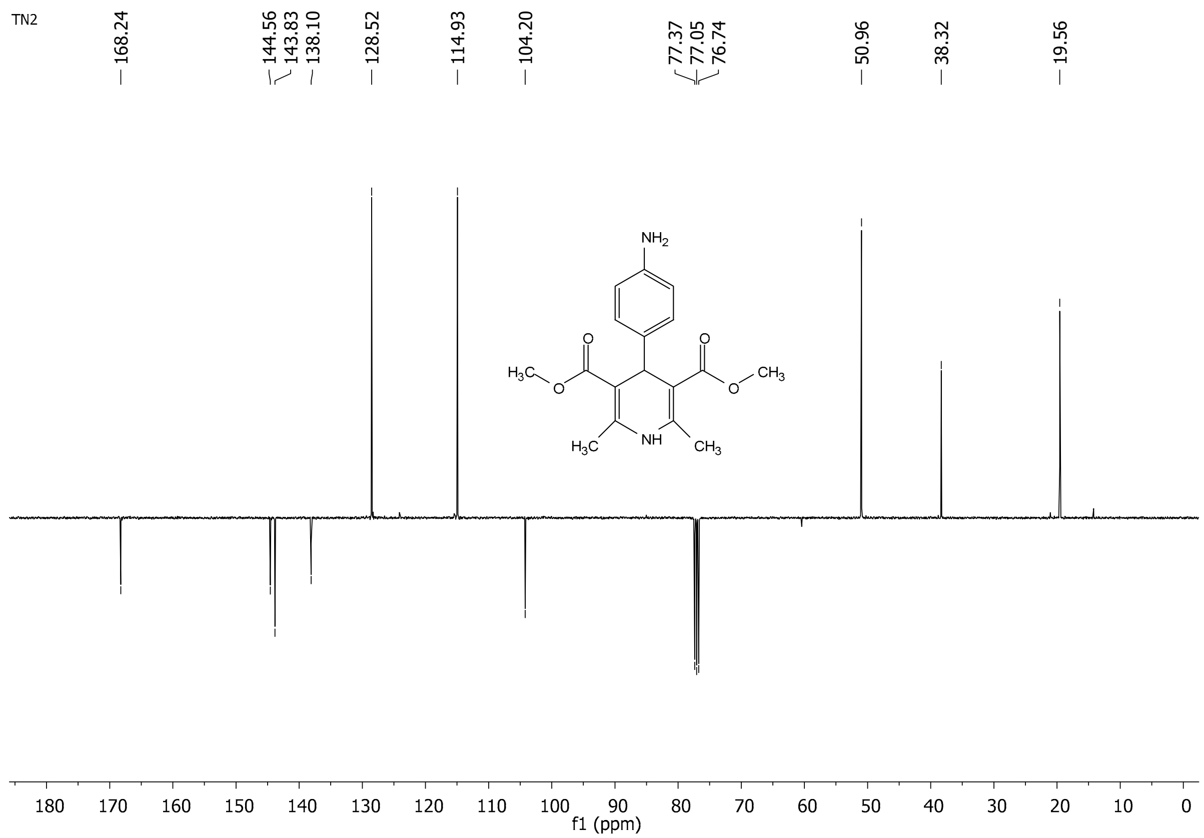

^{1}H NMR spectrum of compound **3b**

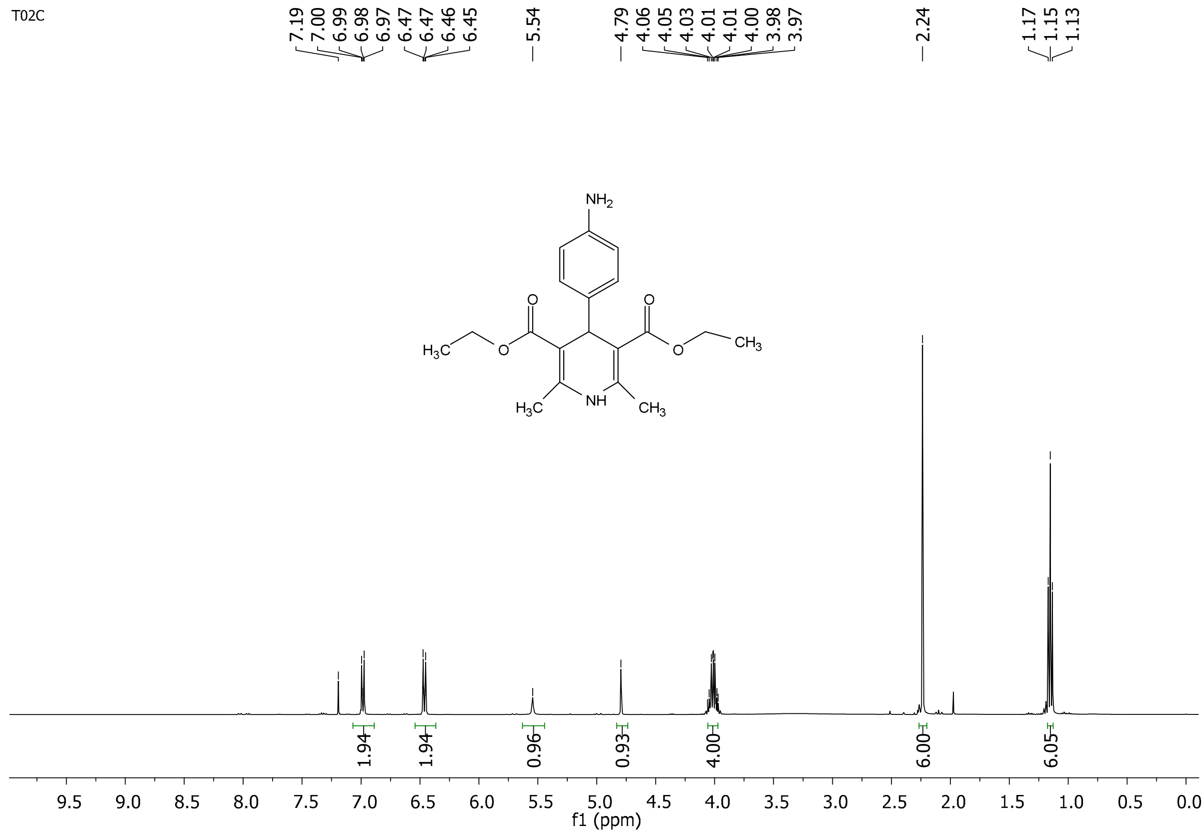

^{13}C NMR spectrum of compound **3b**

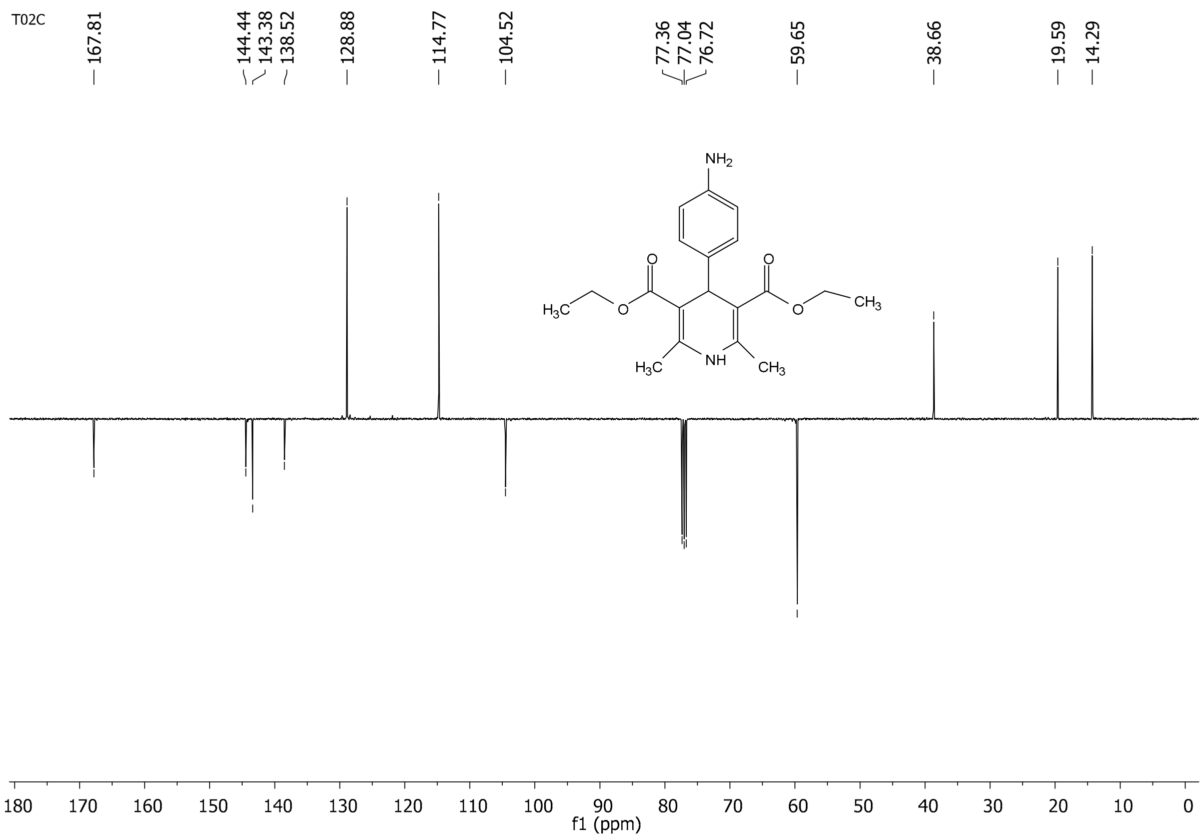

^{1}H NMR spectrum of compound **3c**

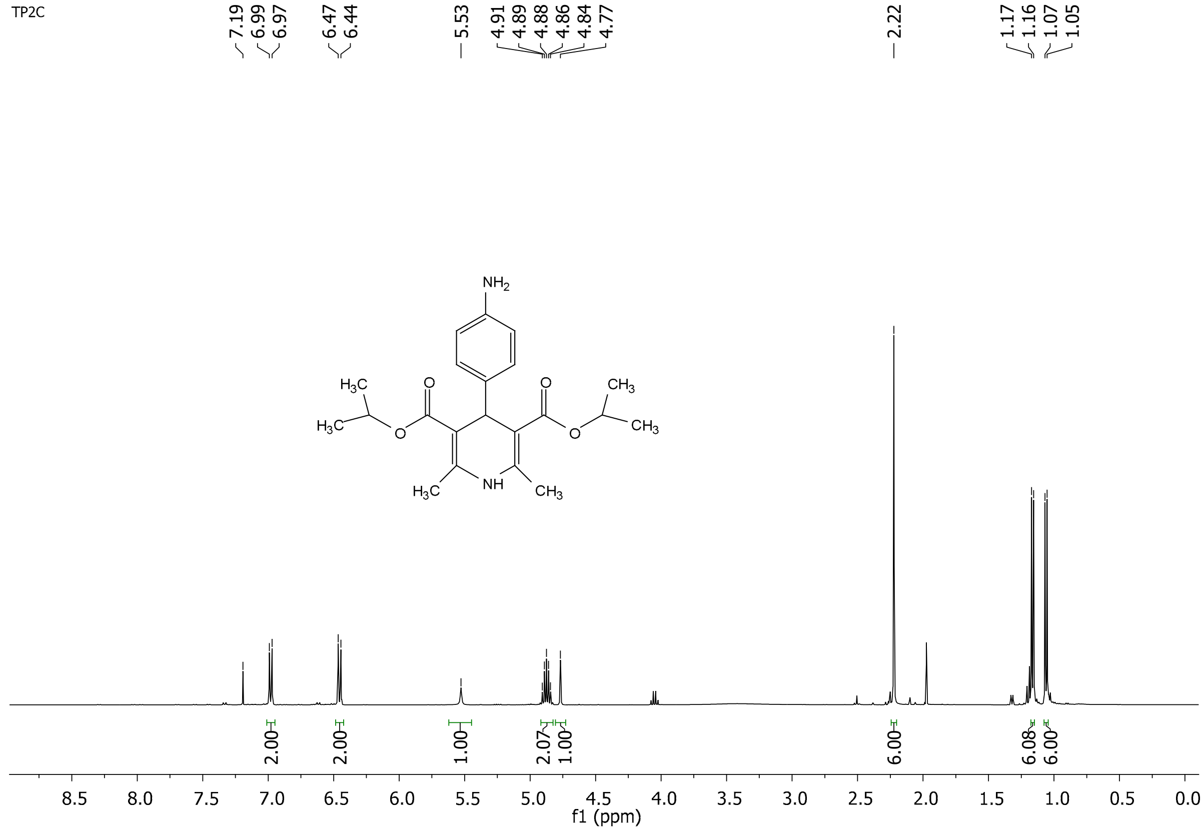

^{13}C NMR spectrum of compound **3c**

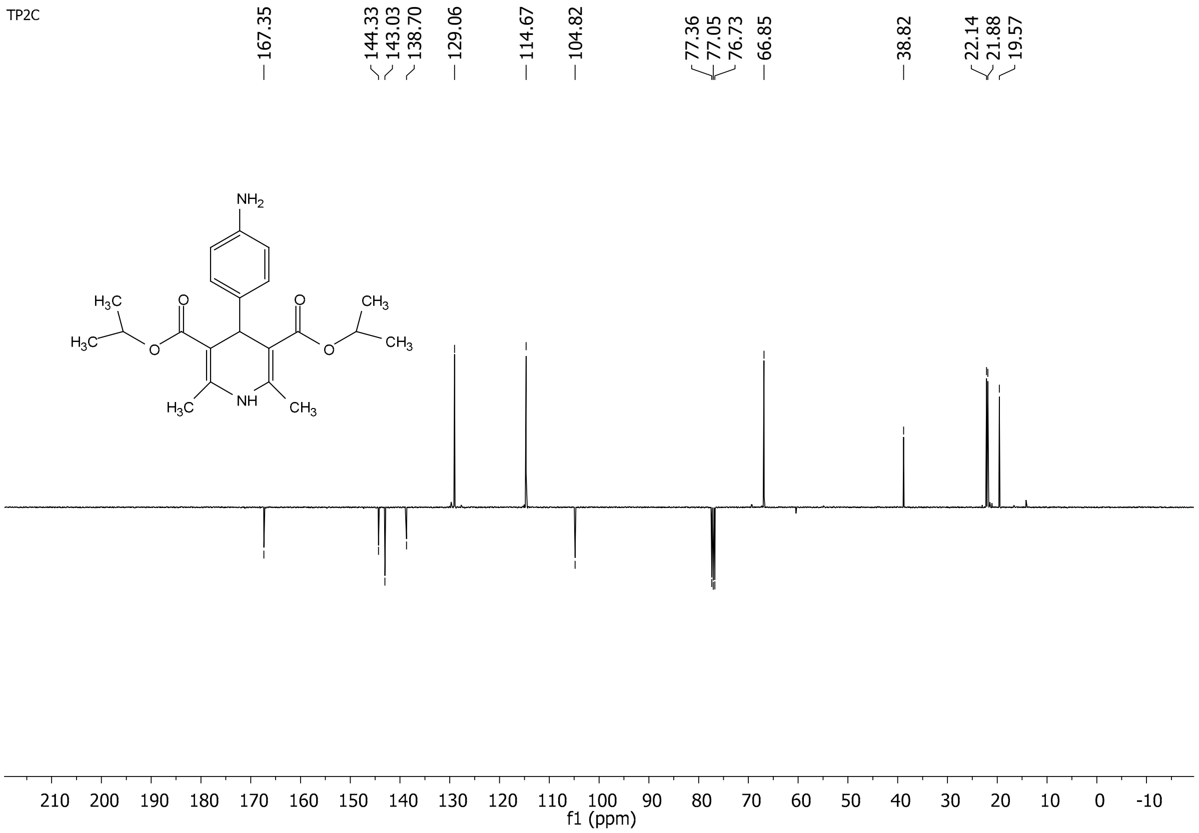

^{1}H NMR spectrum of compound **4a**

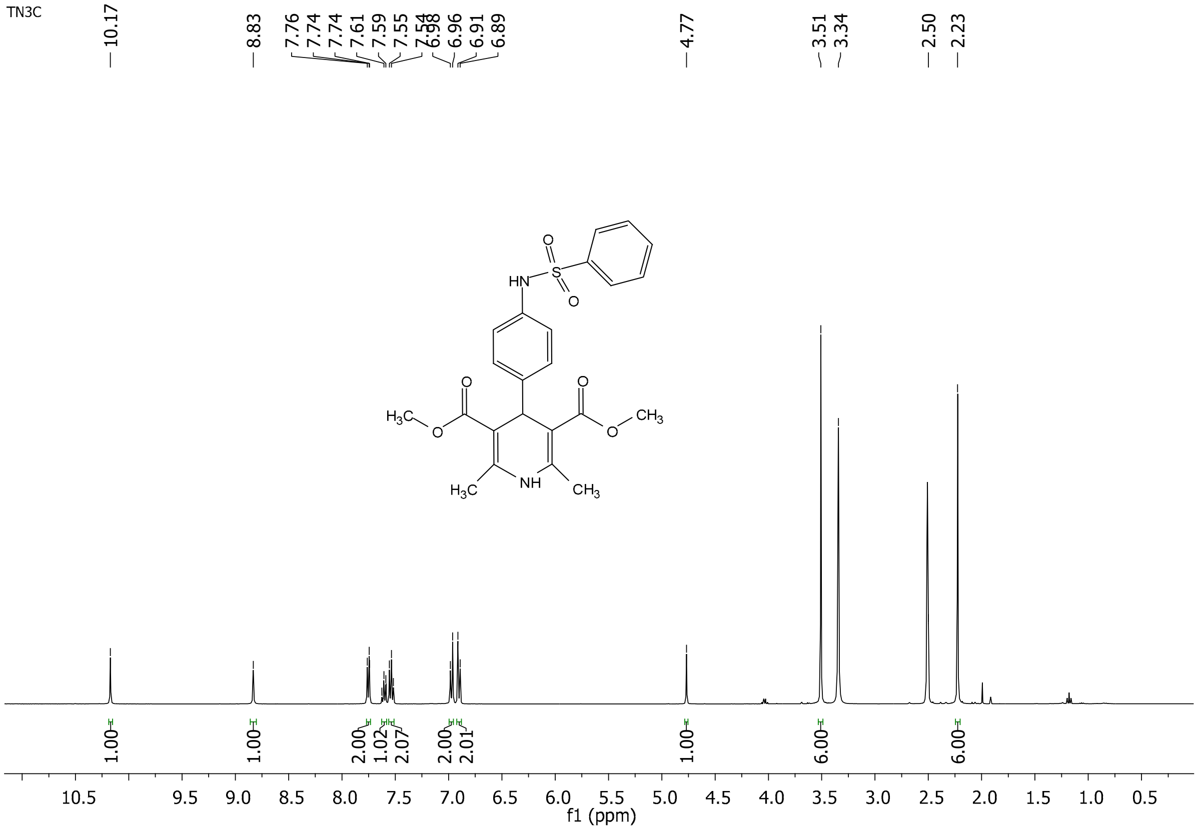

^{13}C NMR spectrum of compound **4a**

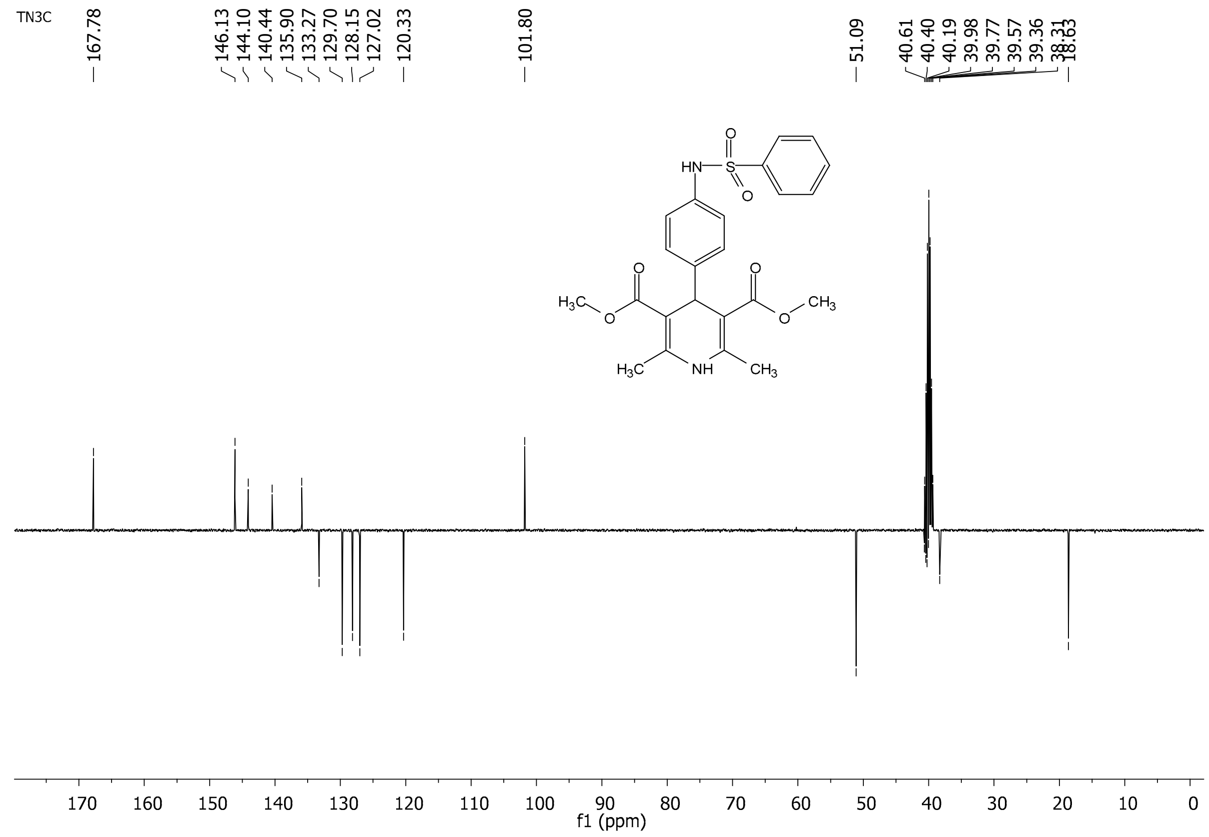

^{1}H NMR spectrum of compound **4b**

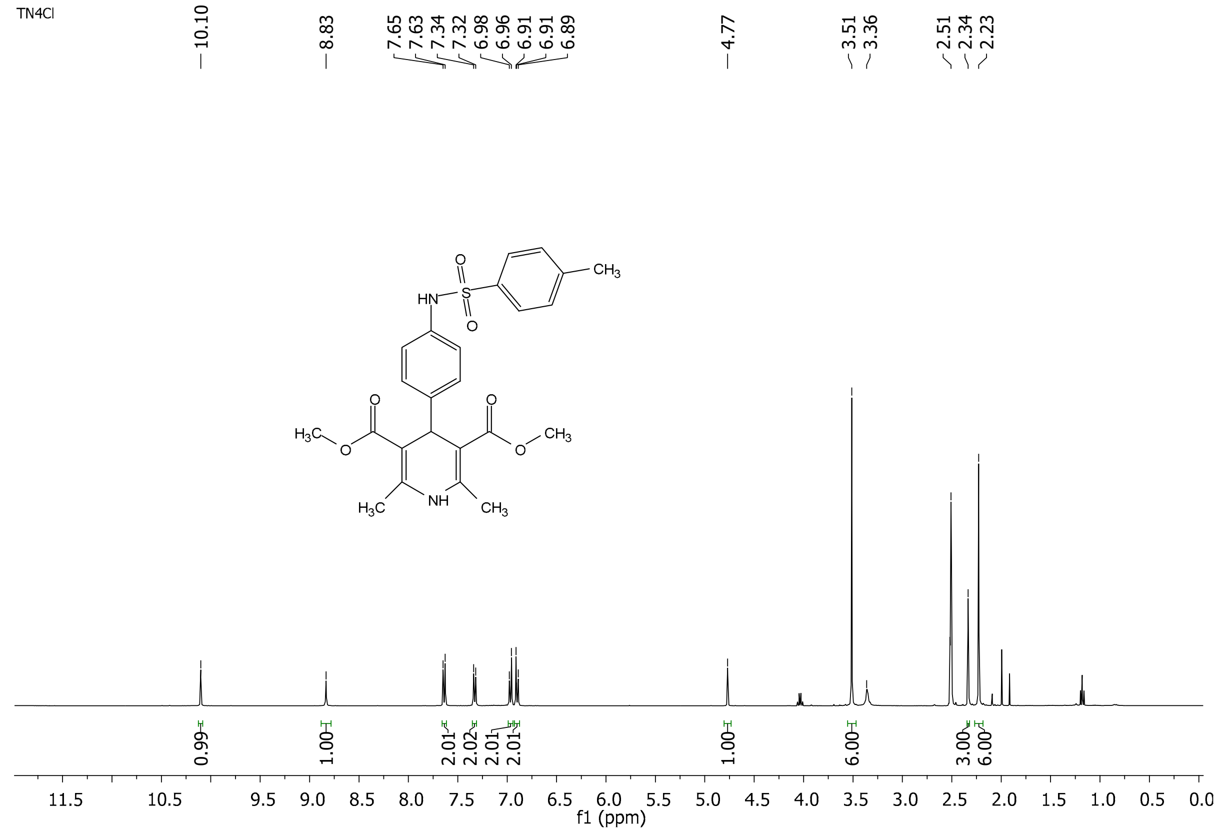

^{13}C NMR spectrum of compound **4b**

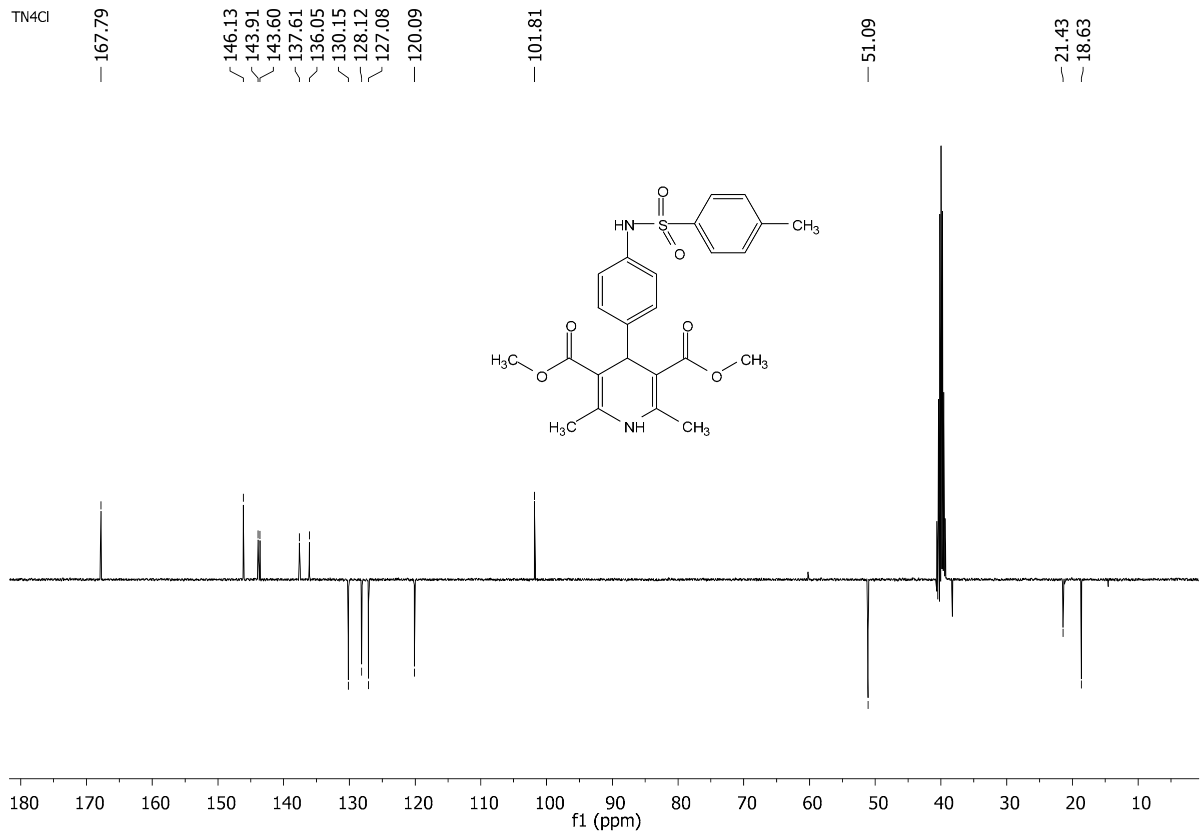

^{1}H NMR spectrum of compound **4c**

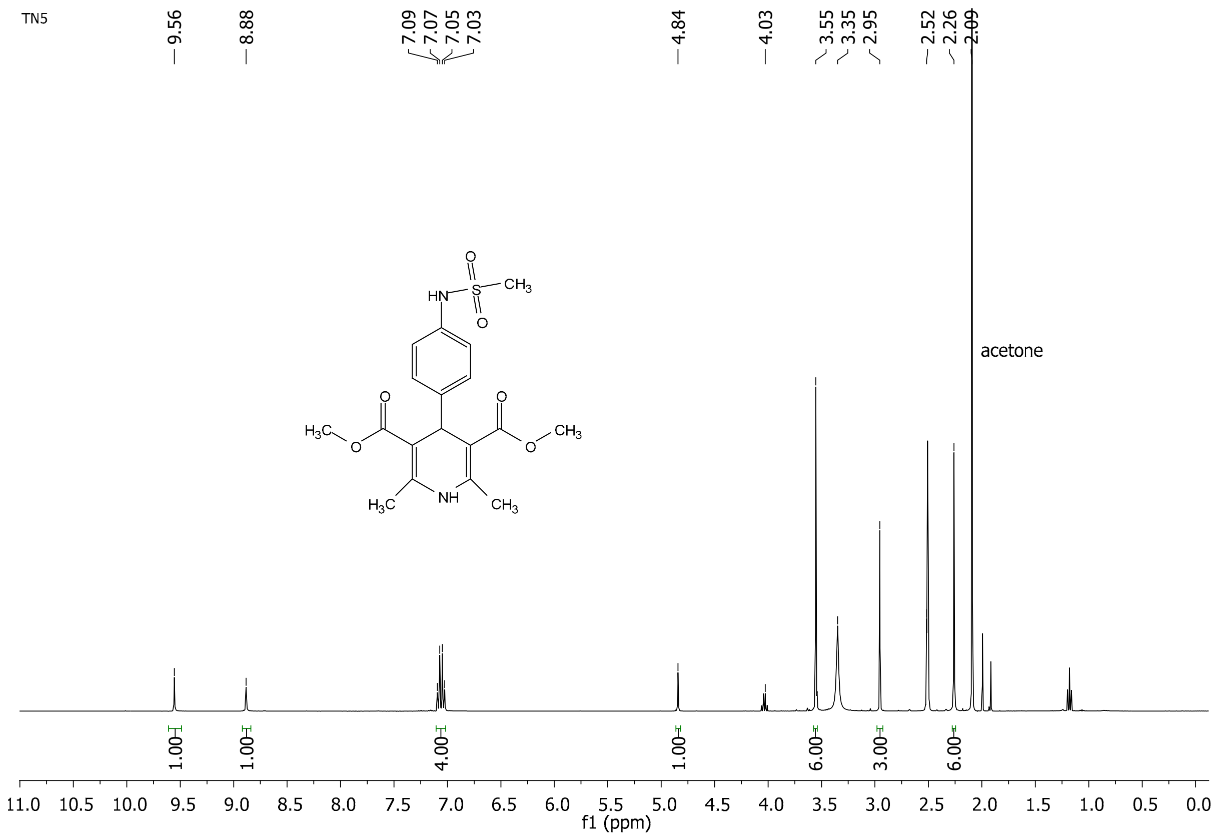

^{13}C NMR spectrum of compound **4c**

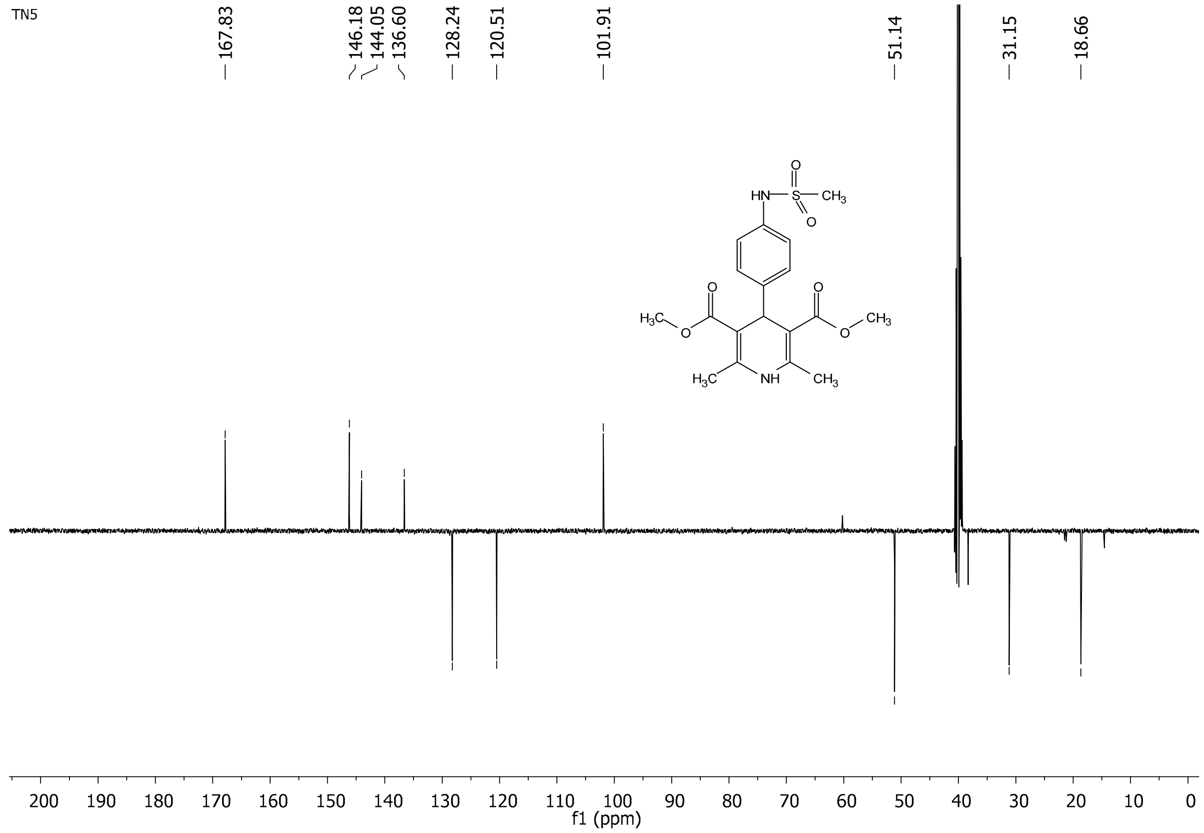

^{1}H NMR spectrum of compound **4d**

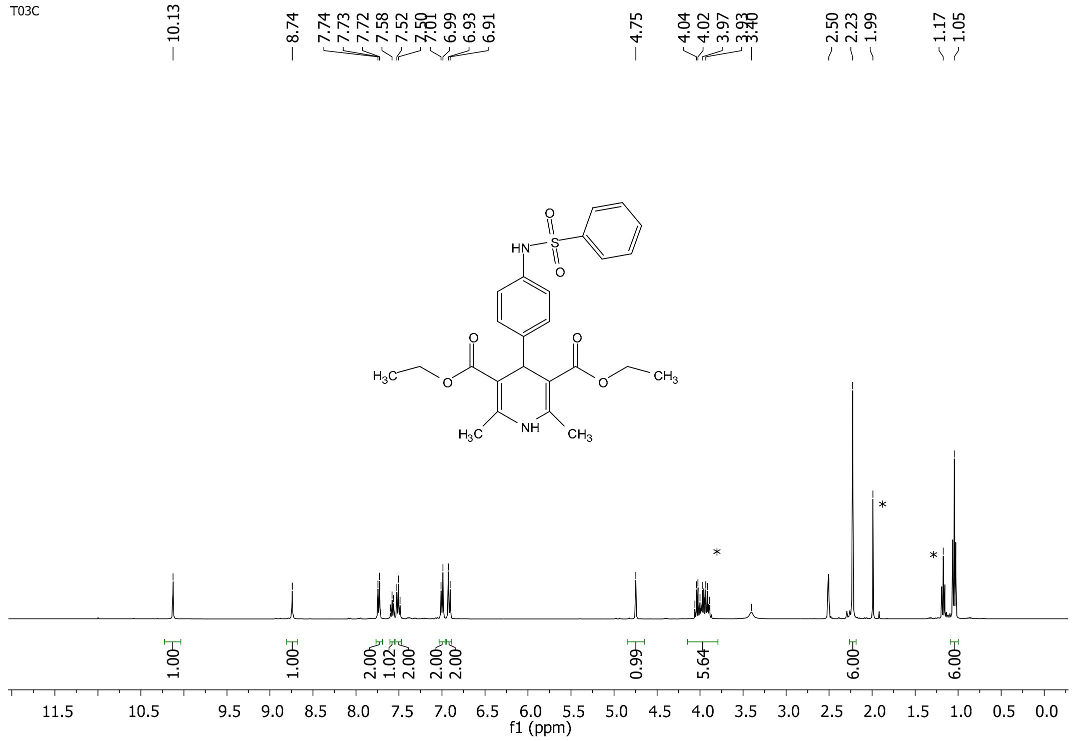

solvent ethyl acetate *

^{13}C NMR spectrum of compound **4d**

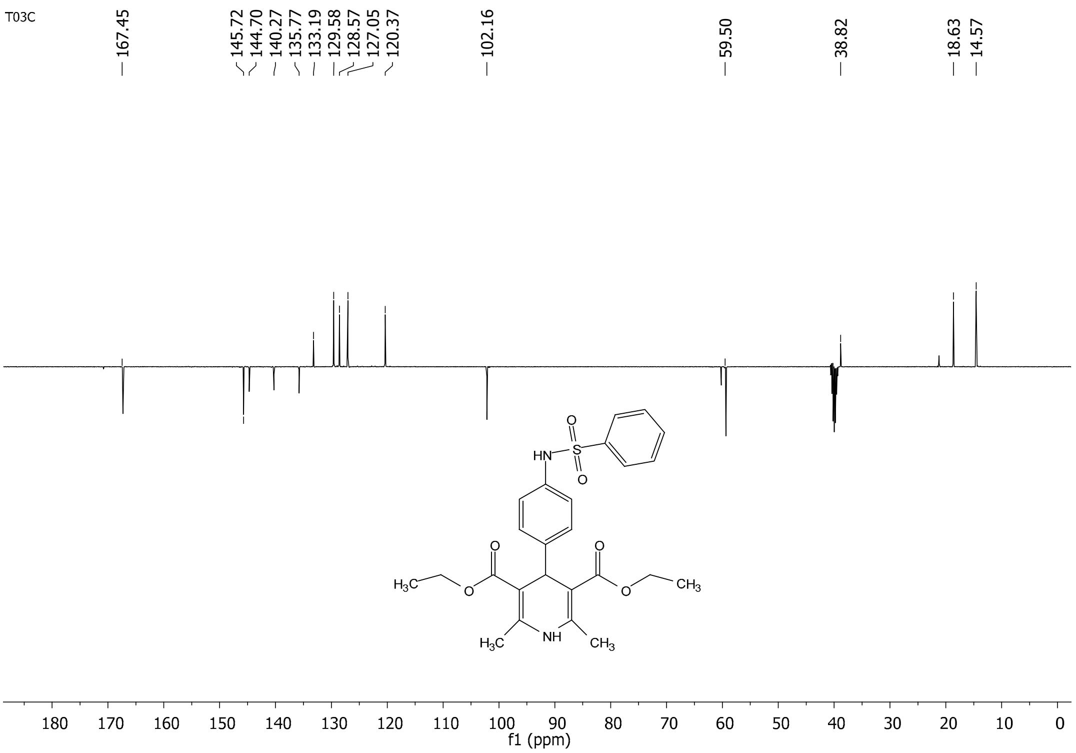

^{1}H NMR spectrum of compound **4e**

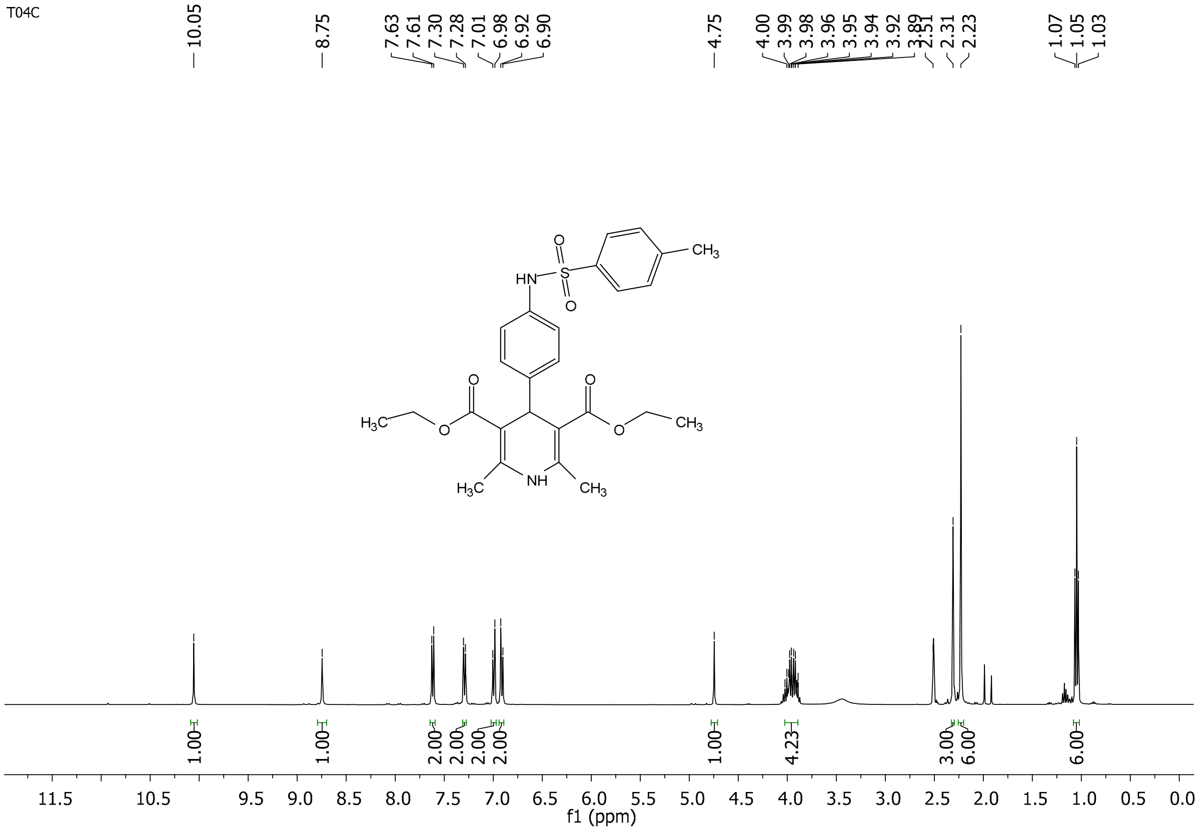

^{13}C NMR spectrum of compound **4e**

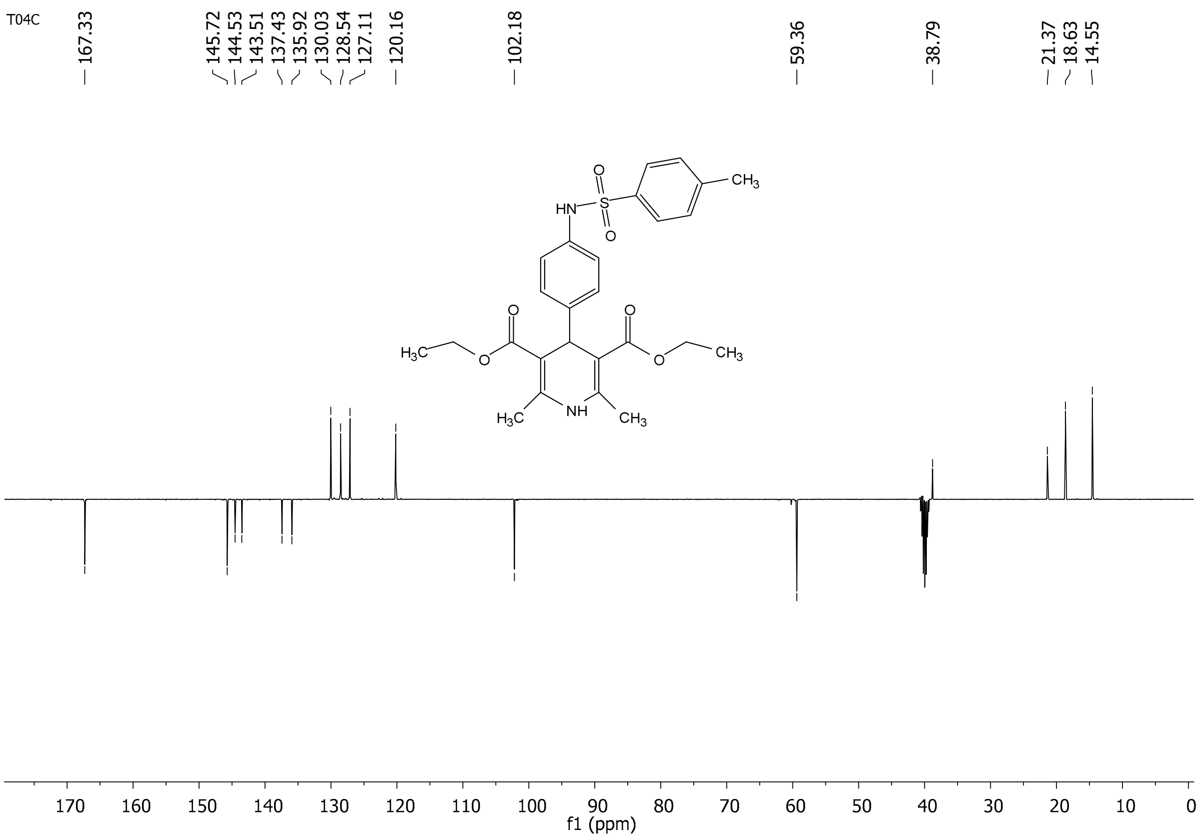

^{1}H NMR spectrum of compound **4f**

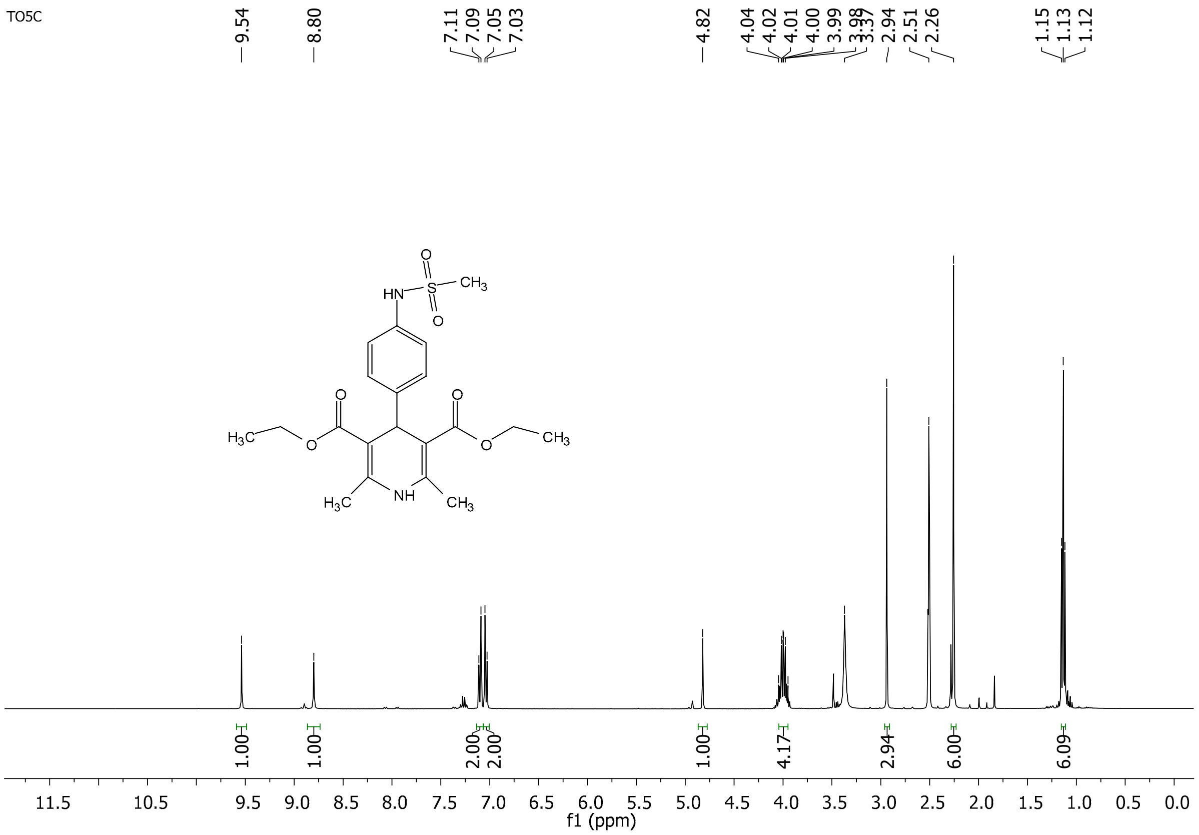

^{13}C NMR spectrum of compound **4f**

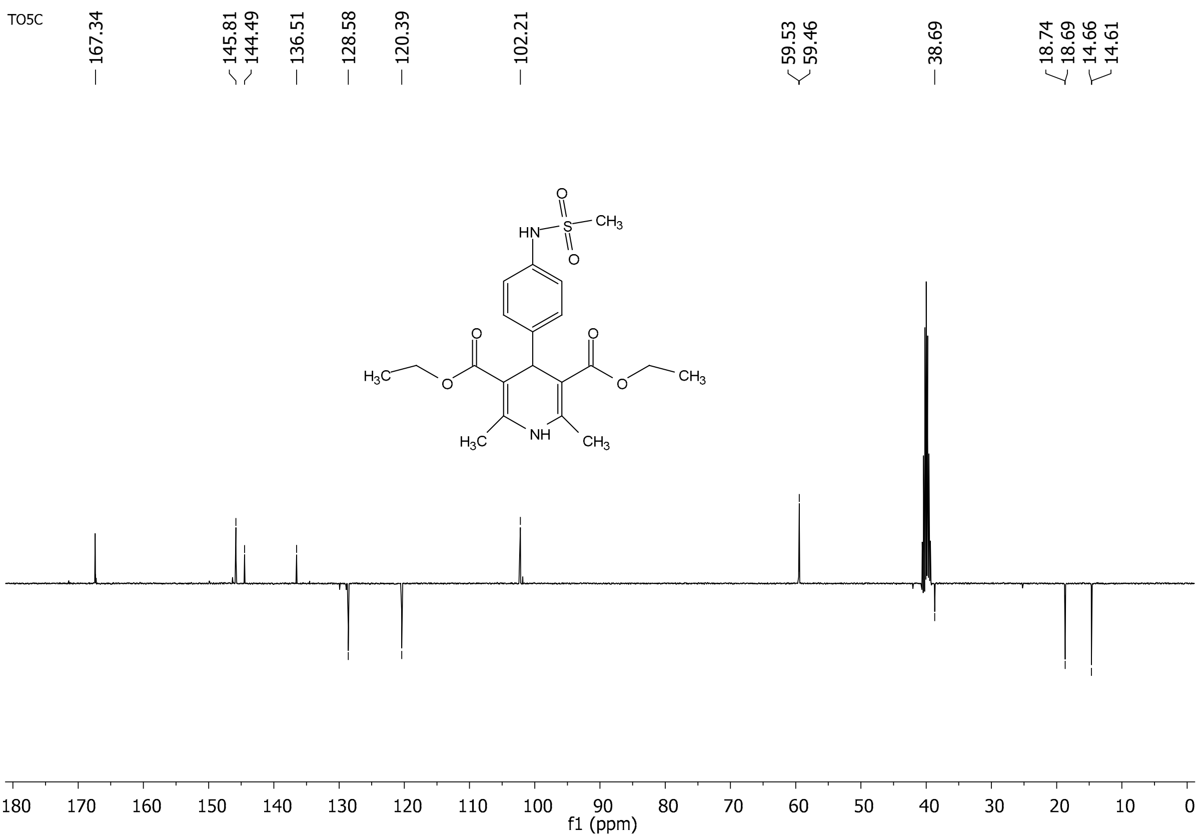

^{1}H NMR spectrum of compound **4g**

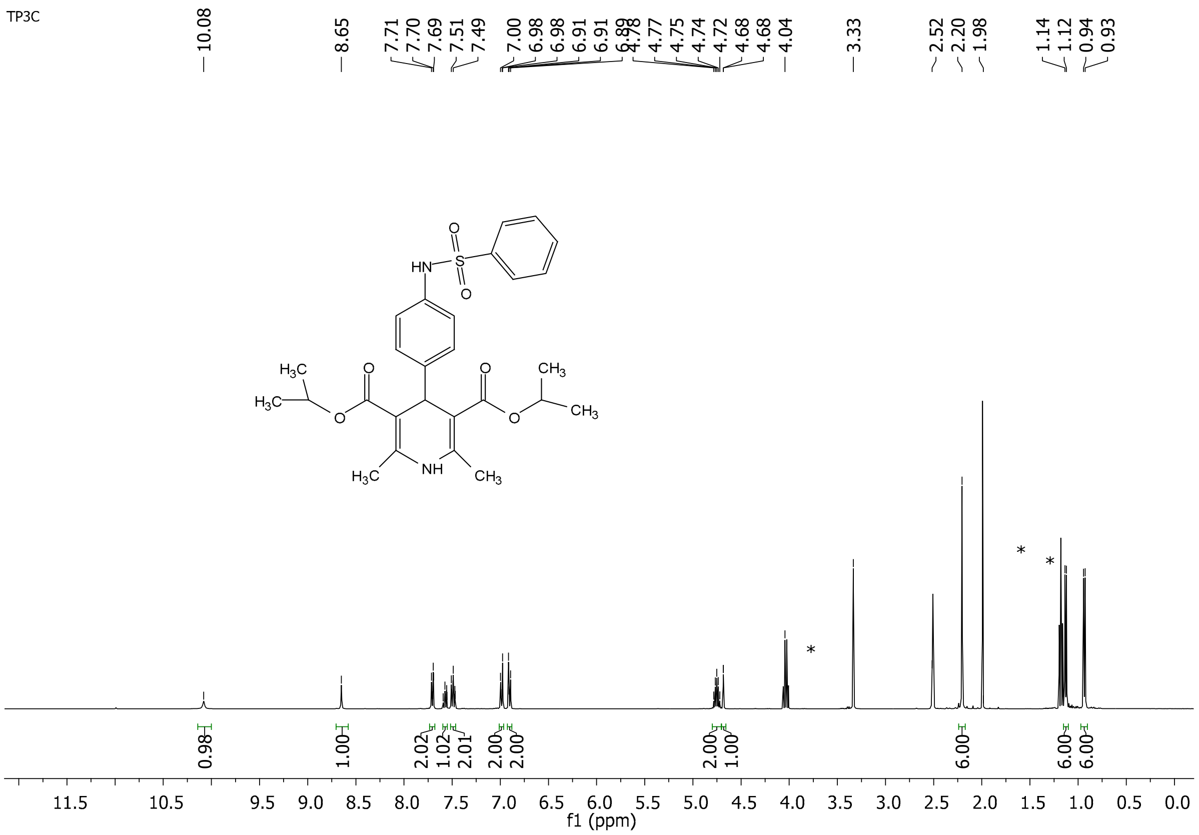

Ethyl aetate *

^{13}C NMR spectrum of compound **4g**

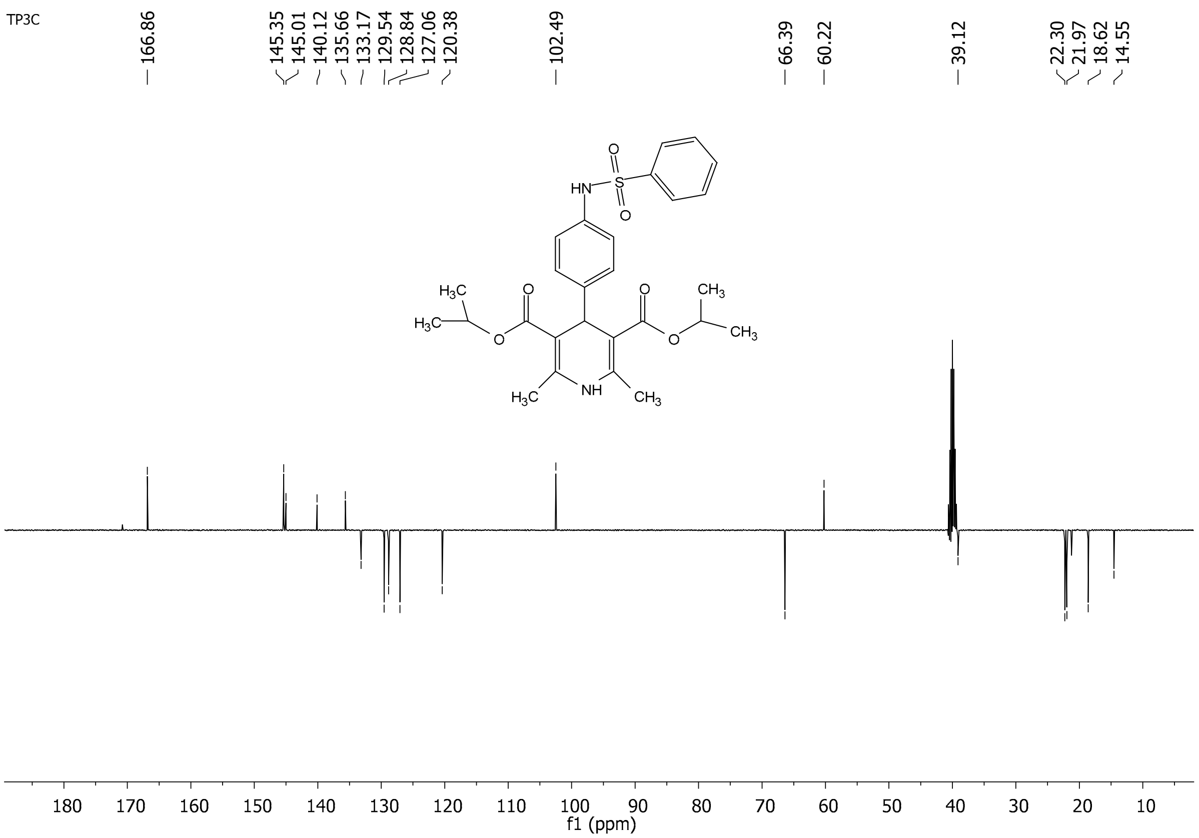

^{1}H NMR spectrum of compound **4h**

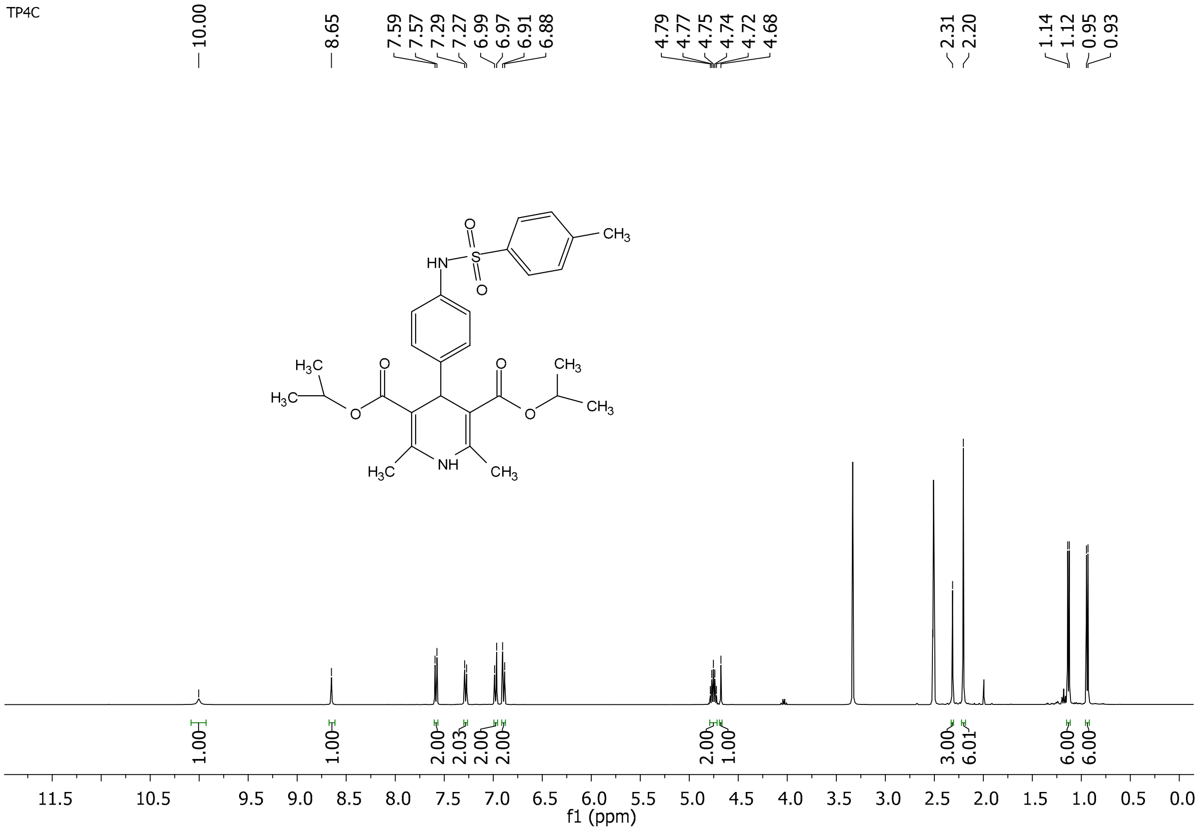

^{13}C NMR spectrum of compound **4h**

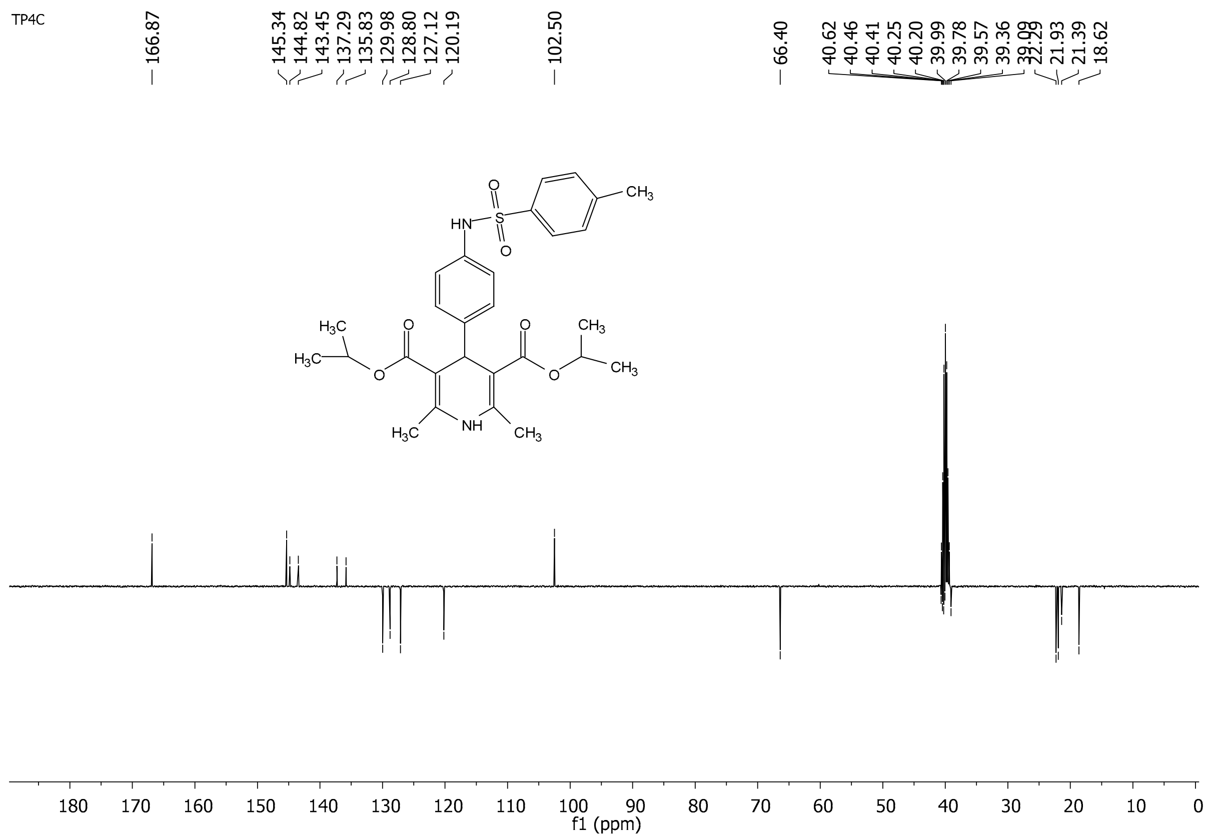

^{1}H NMR spectrum of compound **4i**

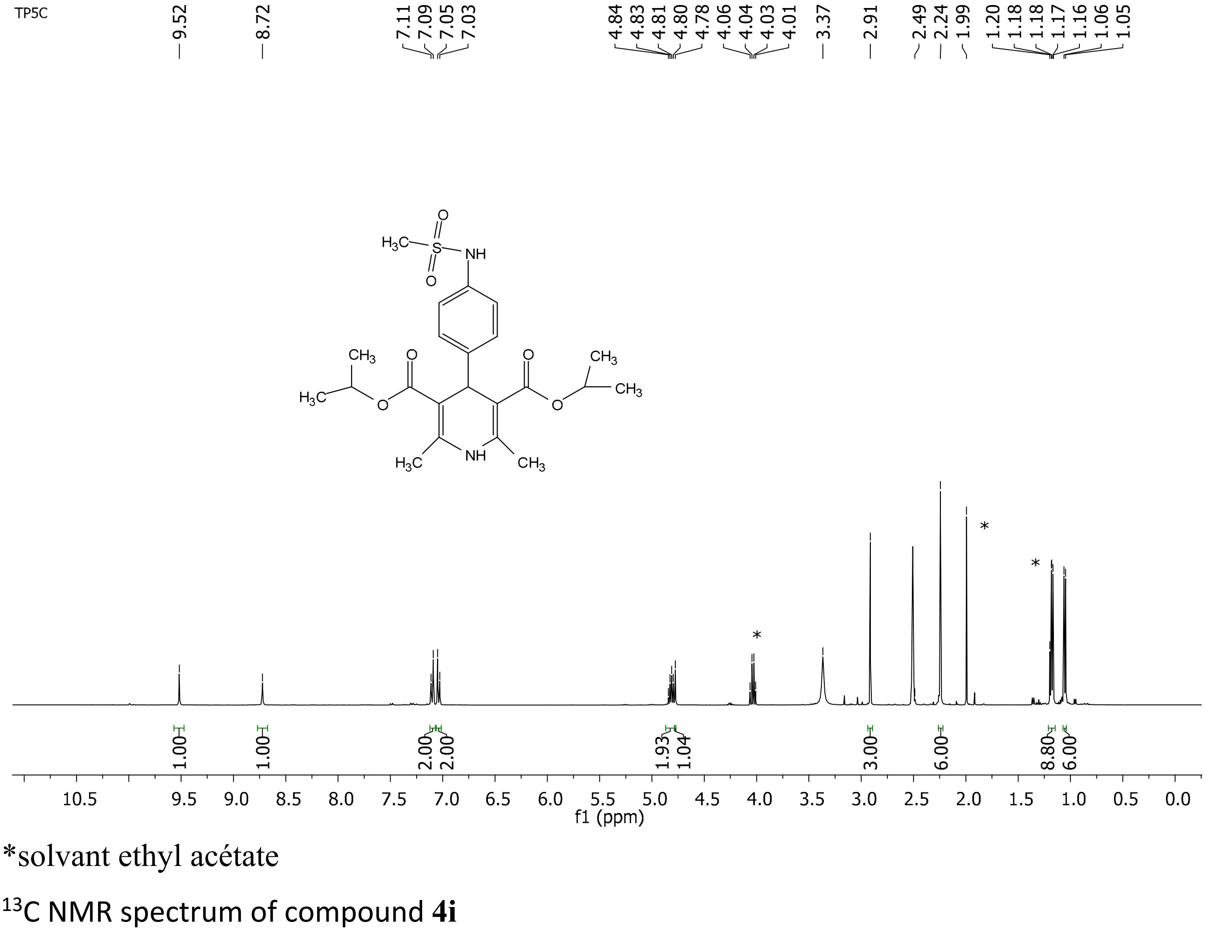

*solvant ethyl acétate

^{13}C NMR spectrum of compound **4i**

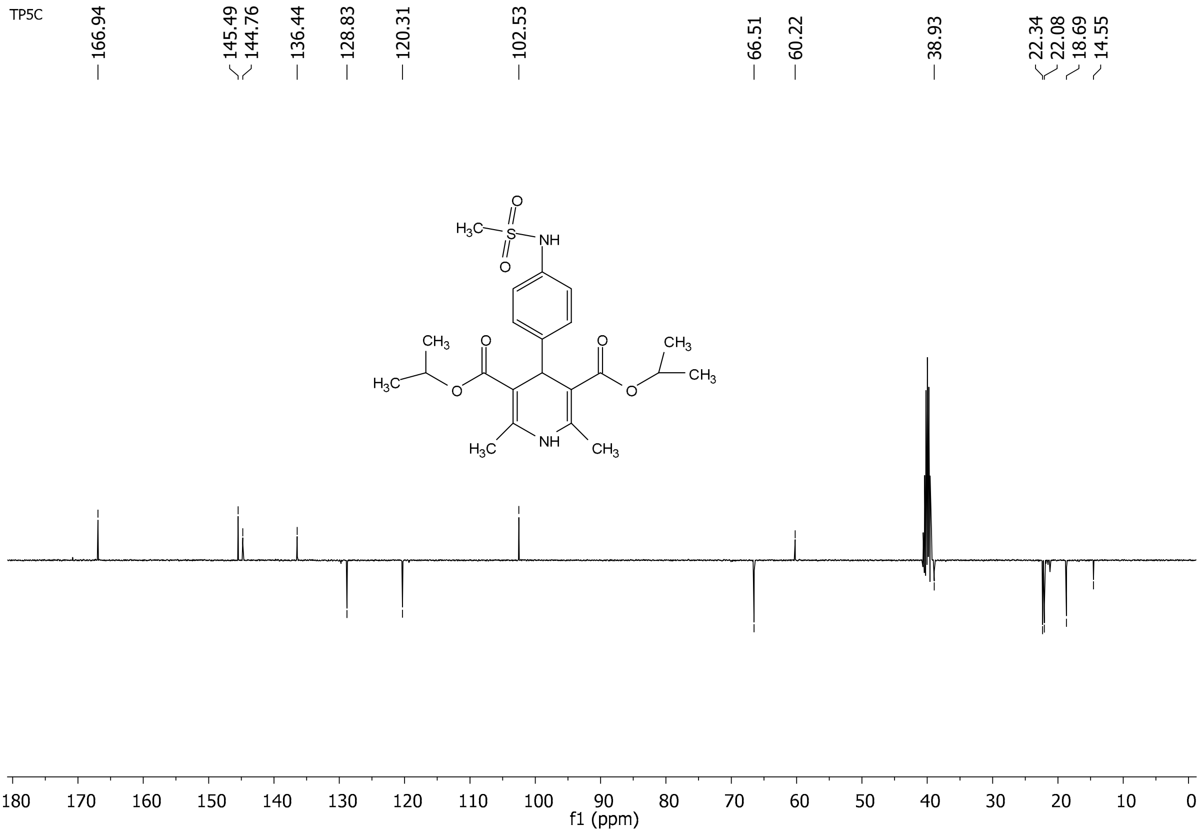

Inhibition of *Ee*AChE and eqBuChE

Inhibitory activity assessment of the TFAHs on ChEs was performed following the spectrophotometric method of Ellman using purified AChE from *Electrophorus electricus* (Type V-S, Sigma-Aldrich) or BuChE from horse serum (lyophilized powder, Sigma-Aldrich). Enzymes were first dissolved in 0.1 M phosphate buffer (pH= 8.0) and then aliquoted in small vials. Compounds stock solutions in DMSO (10 mM) were further diluted with DMSO to prepare nine serial dilutions of each compound. The reaction occurs in a final volume of 3 mL of a 0.1 M phosphate-buffered solution at pH= 8.0, containing 5,5'-dithiobis-2-nitrobenzoic acid (DTNB, 2625 µL, 0.35 mM, final concentration), *Ee*AChE (29 µL, 0.035 U/mL, final concentration) or *eq*BuChE (60 µL, 0.05 U/mL, final concentration), tested compound (3 µL, 0.1-10 µM, final concentrations). Inhibition curves were built by pre-incubating this blend at room temperature with six concentrations of each compound for 10 min. A control with no compound was always present to determine the percent of enzymatic activity. After this pre-incubation period, acetylthiocholine iodide (105 µL, 0.35 mM, final concentration) or butyrylthiocholine iodide (150 µL, 0.5 mM, final concentration) was added, allowing 15 min of additional incubation time, where the DTNB produces the

yellow anion 5-thio-2-nitrobenzoic acid as an indicator of enzymatic activity. After 15 min, absorbances were measured at 412 nm in a spectrophotometer plate reader (iEMS Reader MF, Labsystems). Color generation would be reduced as the compound inhibits the enzymes. IC_{50} values were determined graphically from log concentration–% inhibition curves (GraphPad Prism 5.3 software, GraphPad Software Inc.). Data are expressed as means ± SEM of at least three different experiments in quadruplicate.

Chromatogram of 4a

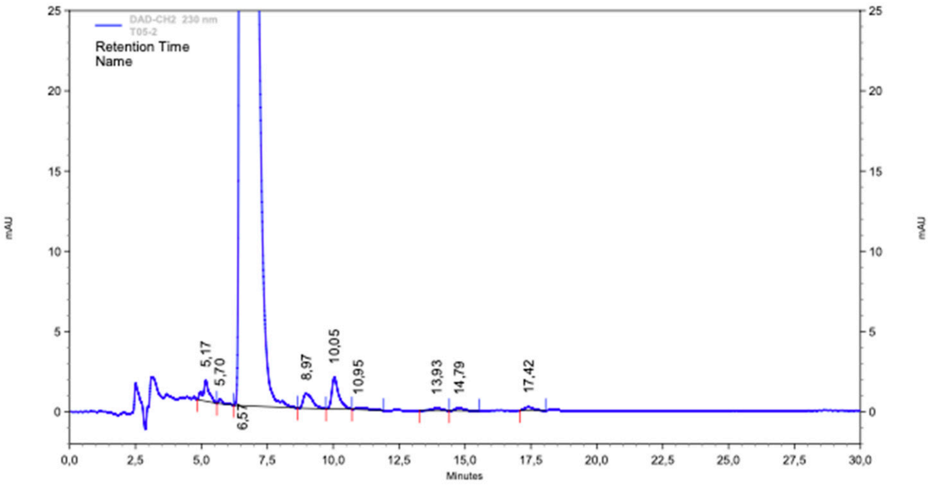

DAD-CH2 230 nm
Results

N°	Temps (min)	Surface	Int. code	Area Percent
1	5,17	82080	vv	0,20
2	5,70	12475	vv	0,03
3	6,57	39955450	vv	99,02
4	8,97	89886	vv	0,22
5	10,05	142768	vv	0,35
6	10,95	13559	vv	0,03
7	13,93	16778	vv	0,04
8	14,79	17187	vv	0,04
9	17,42	20800	vv	0,05

Chromatogram of 4f

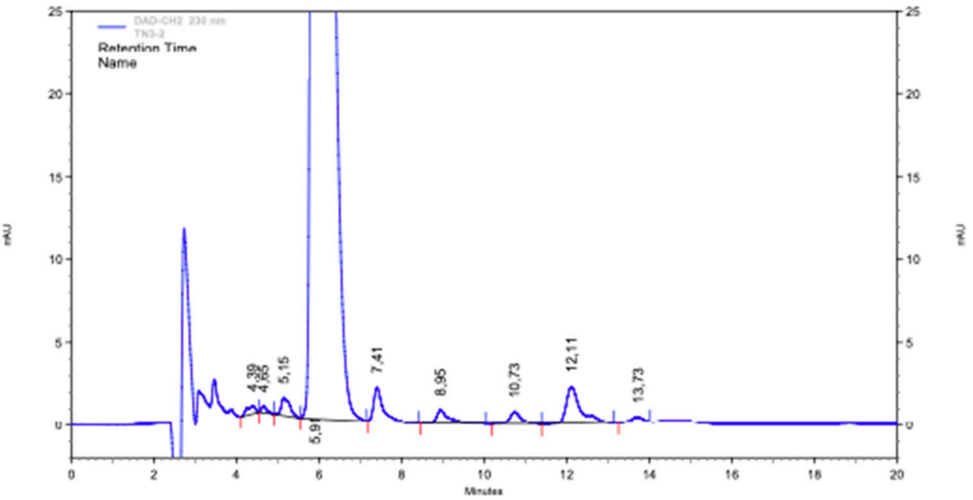

DAD-CH2 230 nm

Results

N°	Temps (min)	Surface	Int. code	Area Percent
1	4,39	30068	vv	0,09
2	4,65	15196	vv	0,05
3	5,15	64524	vv	0,20
4	5,91	31373300	vv	98,06
5	7,41	134071	vv	0,42
6	8,95	71946	vv	0,22
7	10,73	62131	vv	0,19
8	12,11	218469	vv	0,68
9	13,73	24179	vv	0,08
